# *Lactobacillus helveticus*-Fermented Milk Whey Suppresses Melanin Production by Inhibiting Tyrosinase through Decreasing MITF Expression

**DOI:** 10.3390/nu12072082

**Published:** 2020-07-14

**Authors:** Nobutomo Ikarashi, Natsuko Fukuda, Makiba Ochiai, Mami Sasaki, Risako Kon, Hiroyasu Sakai, Misaki Hatanaka, Junzo Kamei

**Affiliations:** 1 Department of Biomolecular Pharmacology, Hoshi University, 2-4-41 Ebara, Shinagawa-ku, Tokyo 142-8501, Japan; s151206@hoshi.ac.jp (N.F.); s161077@hoshi.ac.jp (M.O.); s161131@hoshi.ac.jp (M.S.); r-kon@hoshi.ac.jp (R.K.); sakai@hoshi.ac.jp (H.S.); kamei@hoshi.ac.jp (J.K.); 2 Asahi Calpis Wellness Co., Ltd., 2-4-1 Ebisu-minami, Shibuya-ku, Tokyo 150-0022, Japan; misaki.hatanaka@asahicalpis-w.co.jp

**Keywords:** whey, *Lactobacillus helveticus*, melanin, α-melanocyte-stimulating hormone, tyrosinase, tyrosinase-related protein 1, dopachrome tautomerase, microphthalmia-associated transcription factor, cosmetics

## Abstract

Whey obtained from milk fermented by the *Lactobacillus helveticus* CM4 strain (LHMW) has been shown to improve skin barrier function and increase skin-moisturizing factors. In this study, we investigated the effects of LHMW on melanin production to explore the additional impacts of LHMW on the skin. We treated mouse B16 melanoma cells with α-melanocyte-stimulating hormone (α-MSH) alone or simultaneously with LHMW and measured the amount of melanin. The amount of melanin in B16 cells treated with α-MSH significantly increased by 2-fold compared with that in control cells, and tyrosinase activity was also elevated. Moreover, treatment with LHMW significantly suppressed the increase in melanin content and elevation of tyrosinase activity due to α-MSH. LHMW also suppressed the α-MSH-induced increased expression of tyrosinase, tyrosinase-related protein 1 (TRP1), and dopachrome tautomerase (DCT) at the protein and mRNA levels. Furthermore, the mRNA and protein microphthalmia-associated transcription factor (MITF) expression levels were significantly increased with treatment with α-MSH alone, which were also suppressed by LHMW addition. LHMW suppression of melanin production is suggested to involve inhibition of the expression of the tyrosinase gene family by lowering the MITF expression level. LHMW may have promise as a material for cosmetics with expected clinical application in humans.

## 1. Introduction

Melanin, which is the end product of melanogenesis, is generated in the melanosomes of melanocytes and is an important factor determining the color of human skin, hair, and eyes [1,2]. Melanin is associated with protection of the skin from ultraviolet rays; however, excess melanin production and accumulation on the skin cause pigmentation disorders, such as freckles, skin discoloration, and pigmented age spots [3].

In addition to ultraviolet irradiation, melanin production is induced by various hormones, including α-melanocyte-stimulating hormone (α-MSH) and estrogen [4,5], as well as by environmental stimulation from chemical substances such as theophylline [6,7] and extracellular stimuli, such as cytokines [8]. Melanogenesis is catalyzed by three types of melanocyte-specific enzymes: tyrosinase, tyrosinase-related protein 1 (TRP1), and dopachrome tautomerase (DCT) [9]. Tyrosinase is a rate-limiting enzyme of the melanin production pathway, in which l-tyrosine is converted to l-3,4-dihydroxyphenylalanine (l-DOPA) via hydroxylation. l-DOPA is in turn oxidized to become DOPA quinone [10]. DCT, which is also called TRP2, catalyzes the tautomerization of dopachrome to produce 5,6-dihydroxyindole-2-carboxylic acid (DHICA) [11]. TRP1 also oxidizes DHICA to produce carboxylate indole-quinone [12]. TRP1 and DCT function downstream from tyrosinase in the melanin biosynthesis pathway [2]. Therefore, these enzymes are suitable targets for the development of cosmetics aimed at skin whitening, and such novel materials have been actively explored [13,14].

Milk fermented by the *Lactobacillus helveticus* CM4 strain (LH-fermented milk), a lactic acid bacterium, has been reported to have a hypotensive effect [15,16] and a learning–memory improvement effect [17]. LH-fermented milk cofermented with yeast has been shown to have life-extension [18] and antitumor effects [19]. Moreover, whey obtained from LH-fermented milk (LH-fermented milk whey (LHMW)) was shown to strengthen epidermal barrier function when taken orally and was effective in the prevention of dermatitis [20]. Furthermore, the expression of profilaggrin, which is an important factor for skin moisturizing, was reported to increase when LHMW was added to normal human epidermal keratinocytes [21]. As such, it has been clarified that LHMW has useful effects on the skin, and its application has been gaining attention. To expand the effects of LHMW on the skin, in this study, we investigated the effect of LHMW on melanin production. Specifically, we treated B16 cells, a mouse melanocyte cell line, with α-MSH with and without LHMW to investigate the suppressive effect of LHMW upon induction of melanin production and to explore the underlying molecular mechanism.

## 2. Materials and Methods

### 2.1. Materials

Dulbecco's modified Eagle medium (DMEM) and 2-amino-2-hydroxymethyl-1,3-propanediol (Tris) were purchased from Fujifilm Wako Pure Chemical Co., Ltd. (Osaka, Japan). The cell proliferation reagent water-soluble tetrazolium salt (WST-1) was purchased from Roche (Mannheim, Germany). Bovine serum albumin (BSA) and TRI reagent were purchased from Sigma-Aldrich Corp. (St. Louis, MO, USA). Mouse antihuman tyrosinase (T311) antibody, mouse antihuman TRP1 (G-9) antibody, mouse antihuman TRP2/DCT (C-9) antibody, and mouse antihuman microphthalmia-associated transcription factor (MITF; D-9) antibody were purchased from Santa Cruz Biotechnology, Inc. (Santa Cruz, CA, USA). Rabbit anti-β-actin antibody was purchased from BioLegend (San Diego, CA, USA). Donkey antimouse IgG-HRP antibody, donkey antirabbit IgG-HRP antibody, and enhanced chemiluminescence (ECL) prime Western blotting detection reagents were purchased from GE Healthcare (Waukesha, WI, USA). A high-capacity cDNA synthesis kit was purchased from Applied Biosystems (Foster City, CA, USA). SsoAdvanced Universal SYBR Green Supermix was purchased from Bio-Rad Laboratories (Hercules, CA, USA).

### 2.2. Preparation of Fermented Milk Whey

Fermented milk was prepared as reported previously [21]. In brief, reconstituted, pasteurized 9% (*w*/*w*) skim milk solution was fermented with the *L. helveticus* CM4 strain at 35 °C for 24 h. This sample was separated into the whey fraction by ultrafiltration (MW < 5 kDa).

### 2.3. Cell Culture

B16 mouse melanoma (Riken Cell Bank, Ibaraki, Japan) cells were maintained in DMEM containing 100 U/mL penicillin G potassium, 100 μg/mL streptomycin, and 10% FBS. B16 cells were seeded in a plate at a density of 2.5 × 10^4^ cells/cm^2^ and incubated in a CO_2_ incubator for 2 days. α-MSH (final concentration: 50 nM) alone or simultaneously with LHMW (final concentration: 1–5%) was added to B16 cells and cultured.

### 2.4. WST-1 Assay

B16 cells seeded in a 96-well plate were treated with LHMW and cultured for 48 h. After removing the medium and washing the cells with phosphate-buffered saline (PBS), WST-1 reagent was added to the cells. After incubation for 4 h, the absorbance at 450 nm and 620 nm was measured using a microplate reader (MTP-450 microplate reader, Corona Electric Co., Ltd., Ibaraki, Japan).

### 2.5. Measurement of Melanin Content

The amount of melanin was measured according to a previously described method, with slight modifications [22,23]. Briefly, α-MSH alone or simultaneously with LHMW was added to B16 cells seeded in a 6-well plate and cultured for 48 h. After B16 cells were washed with PBS, 500 μL of a 1 M NaOH solution was added to each well. The cells were collected and then incubated at 80 °C for 60 min. Absorbance at 415 nm was measured using a microplate reader. Melanin content was normalized by protein content and expressed as a percentage of the control.

### 2.6. Measurement of Tyrosinase Activity

Tyrosinase activity was measured according to a previously described method, with slight modifications [24,25]. α-MSH alone or simultaneously with LHMW was added to B16 cells seeded in a 6-well plate and cultured for 24 h. After B16 cells were washed with PBS, 200 μL of PBS containing 1% Triton X-100 was added to each well. The cells were collected and homogenized with an ultrasonic homogenizer (Handy Sonic, TOMY SEIKO Co., Ltd., Tokyo, Japan) on ice and then centrifuged (11,000× *g*, 20 min, 4 °C). Then, 100 μL of PBS containing 0.1% L-DOPA was added to 30 μL of the supernatant, and the mixture was incubated at 37 °C for 30 min. Absorbance at 492 nm was measured using a microplate reader. Tyrosinase activity was normalized by protein content and expressed as a percentage of the control.

### 2.7. Real-Time PCR

α-MSH alone or simultaneously with LHMW was added to B16 cells seeded in a 6-well plate and cultured for 3 h or 24 h. After washing B16 cells with PBS, total RNA was extracted using TRI reagent. Total RNA was used to calculate the RNA concentration and confirm the purity by measuring the absorbance at 260 nm and 280 nm with a microspectrophotometer (NanoDrop Lite Spectrophotometer, Thermo Fisher Scientific, Waltham, MA, USA). cDNA was synthesized from RNA using a high-capacity cDNA synthesis kit. Real-time PCR was performed to detect the expression of each gene using the specific primers shown in Table 1. The mRNA expression level was normalized using the housekeeping gene glyceraldehyde-3-phosphate dehydrogenase (GAPDH).

### 2.8. Real-Time PCR Preparation of Samples for Western Blotting

α-MSH alone or simultaneously with LHMW was added to B16 cells seeded in a 6-well plate and cultured for 3 h or 24 h. After washing B16 cells with PBS, 500 μL of RIPA buffer was added, and the cells were collected using a cell scraper. The cell suspension solution was placed on ice for 30 min, homogenized by an ultrasonic homogenizer (Handy Sonic, TOMY SEIKO Co., Ltd.), and centrifuged (15,000× *g*, 15 min, 4 °C). The obtained supernatant was used as a sample solution for Western blotting.

### 2.9. Western Blotting

The protein concentration was measured by the bicinchoninic acid (BCA) method. After the addition of an equal volume of loading buffer (100 mM Tris, 20% glycerol, 0.004% bromophenol blue, 4% sodium dodecyl sulfate, and 10% 2-mercaptoethanol; pH 6.8) to the sample solution, the samples were separated using SDS-PAGE. The protein was transferred to a polyvinylidene difluoride membrane and blocked with skim milk solution. The membrane was reacted with the following primary antibodies: mouse antihuman tyrosinase antibody, mouse antihuman TRP1 antibody, mouse antihuman TRP2/DCT antibody, mouse antihuman MITF antibody, or rabbit anti-β-actin antibody. After washing, the membrane was incubated with a secondary antibody, donkey antimouse IgG-HRP antibody or donkey antirabbit IgG-HRP antibody. The antibodies were detected with ECL prime Western blotting detection reagent. The protein signal was visualized using a CCD camera (ImageQuant LAS500, GE Healthcare).

### 2.10. Statistical Analyses

The experimental values are shown as the mean ± standard deviation (SD). Dunnett’s test and Tukey’s test were used for the statistical analyses.

## 3. Results

### 3.1. Effect of LHMW on Melanin Production Stimulated by α-MSH

We investigated the effect of LHMW on melanin production stimulated by α-MSH.

**Figure 1:**
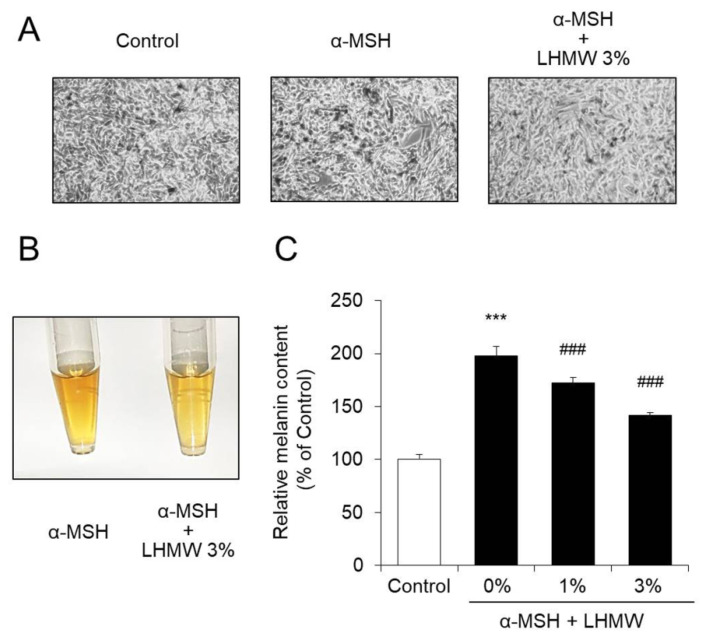
Effect of *Lactobacillus helveticus*-fermented milk whey (LHMW) on melanin production stimulated by α-melanocyte-stimulating hormone (α-MSH). α-MSH alone or simultaneously with LHMW was added to B16 cells and cultured for 48 h (**A**), and the intracellular melanin was eluted (**B**). The amount of melanin was calculated by measuring the absorbance, and the average value of the control was expressed as 100% (**C**) (mean ± SD, *n* = 6, ***; *p* < 0.001 vs. control, ###; *p* < 0.001 vs. vehicle).

When B16 cells were treated with α-MSH and the condition of the cells was observed under a microscope, it was confirmed that melanin production was enhanced. In contrast, cotreatment with LHMW suppressed the amount of melanin stimulated by α-MSH in a concentration-dependent manner. In particular, treatment with 3% LHMW suppressed melanin production induced by α-MSH to approximately the same level as that in the control (Figure 1). The WST-1 assay further showed that treatment with up to 5% LHMW did not affect the cell survival rate of B16 cells (Figure S1).

These results confirmed that LHMW suppressed melanin production stimulated by α-MSH.

### 3.2. Effect of LHMW on Tyrosinase Activity

Tyrosinase is a rate-limiting enzyme of melanogenesis, which is important in the determination of melanin content [13,14]. Therefore, we further investigated whether the suppressive effect of LHMW on melanin production was caused by inhibitory activity on tyrosinase.

**Figure 2:**
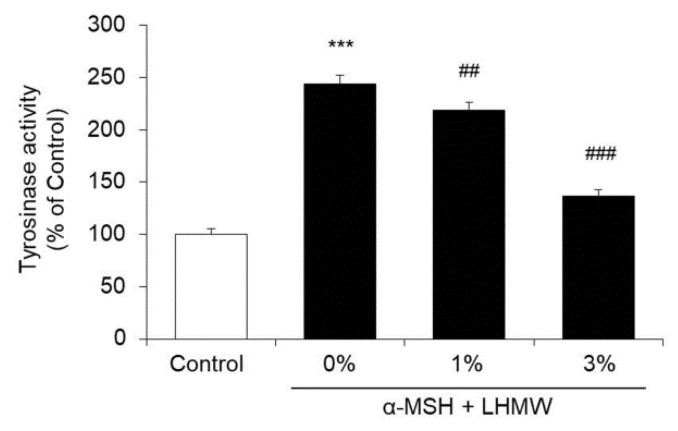
Effect of LHMW on tyrosinase activity. α-MSH alone or simultaneously with LHMW was added to B16 cells and cultured for 24 h. The tyrosinase activity was measured, and the average value of the control was expressed as 100% (mean ± SD, *n* = 6, ***; *p* < 0.001 vs. control, ##; *p* < 0.01, ###; *p* < 0.001 vs. vehicle).

B16 cells treated with α-MSH showed approximately 2-fold higher tyrosinase activity than the control cells, and LHMW suppressed the increase in tyrosinase activity stimulated by α-MSH in a concentration-dependent manner. Specifically, treatment with 3% LHMW resulted in tyrosinase activity similar to that of the control condition (Figure 2).

Therefore, the suppression of melanin production by LHMW was considered to be caused by its inhibitory activity against tyrosinase.

### 3.3. Effects of LHMW on the Protein Expression of Tyrosinase, TRP1, and DCT

We investigated whether the inhibitory effect of LHMW on tyrosinase activity was due to suppression of tyrosinase protein expression. We also analyzed the effect of LHMW on other important proteins for melanin production, including TRP1 and DCT.

**Figure 3:**
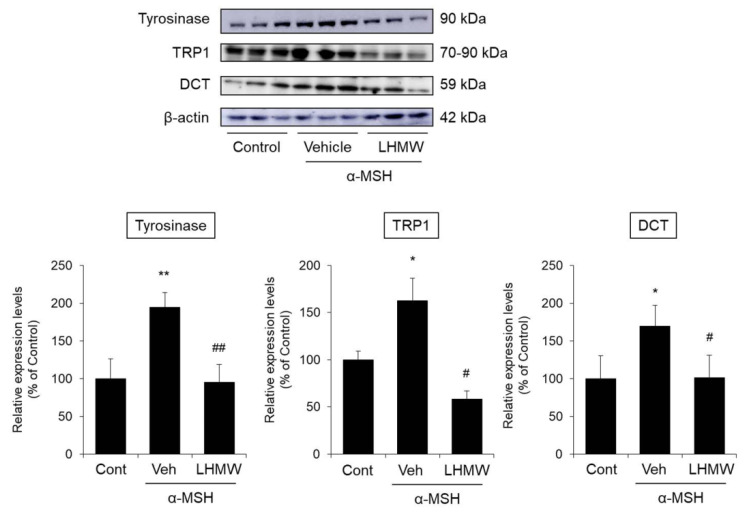
Effects of LHMW on the protein expression of tyrosinase, TRP1, and DCT. α-MSH alone or simultaneously with LHMW was added to B16 cells and cultured for 24 h. The protein expression levels of tyrosinase, TRP1, and DCT were analyzed by Western blotting and normalized to that of β-actin. The average value of the control was expressed as 100% (mean ± SD, *n* = 6, *; *p* < 0.05, **; *p* < 0.01 vs. control, #; *p* < 0.05, ##; *p* < 0.01 vs. vehicle).

The tyrosinase expression level in the α-MSH-treated B16 cells was significantly higher than that in the control cells, and cotreatment with LHMW reduced the protein expression of tyrosinase to approximately the same level as that in the control cells. Similarly, the protein expression levels of TRP1 and DCT increased upon treatment with α-MSH, which were suppressed by LHMW cotreatment to the same level as that in the control cells (Figure 3).

These results indicated that suppressed tyrosinase protein expression was associated with the inhibitory effect of LHMW on tyrosinase activity. LHMW also reduced the levels of TRP1 and DCT expression to suppress melanin production.

### 3.4. Effects of LHMW on Tyrosinase, Trp1, and Dct mRNA Levels

We investigated whether the effect of LHMW in reducing the protein expression levels of tyrosinase, TRP1, and DCT occurred via transcriptional repression.

**Figure 4:**
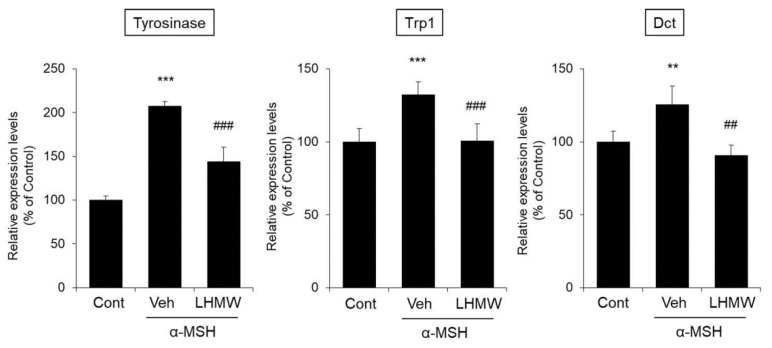
Effects of LHMW on the mRNA expression of tyrosinase, Trp1, and Dct. α-MSH alone or simultaneously with LHMW was added to B16 cells and cultured for 24 h. The mRNA expression levels of tyrosinase, Trp1, and Dct were analyzed by real-time PCR and normalized to that of GAPDH. The average value of the control was expressed as 100% (mean ± SD, *n* = 6, **; *p* < 0.01, ***; *p* < 0.001 vs. control, ##; *p* < 0.01, ###; *p* < 0.001 vs. vehicle).

The expression of tyrosinase mRNA in B16 cells treated with α-MSH was higher than that in the control cells, and LHMW cotreatment reduced tyrosinase mRNA expression to the same level as that in the control cells. The same effects were observed for Trp1 and Dct mRNA levels (Figure 4).

These results clearly demonstrated that LHMW suppressed the transcription of the tyrosinase gene family induced by α-MSH to reduce the protein and mRNA expression levels of tyrosinase, Trp1, and Dct.

### 3.5. Effect of LHMW on MITF Expression

MITF has been shown to regulate the transcription of tyrosinase, Trp1, and Dct [26,27]. Therefore, we investigated whether the reduction in the expression levels of these genes by LHMW was caused by decreasing MITF expression.

**Figure 5:**
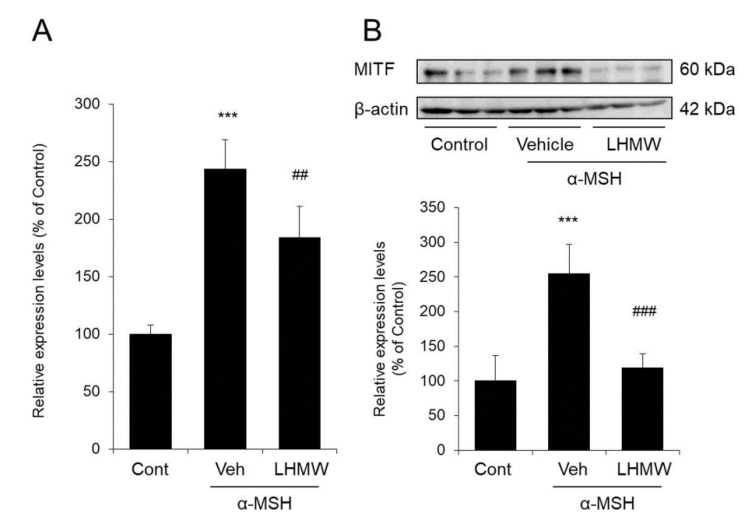
Effect of LHMW on MITF expression. α-MSH alone or simultaneously with LHMW was added to B16 cells and cultured for 3 h. The mRNA (**A**) and protein (**B**) expression levels of MITF were analyzed by real-time PCR and Western blotting and normalized to those of β-actin and GAPDH, respectively. The average value of the control was expressed as 100% (mean ± SD, *n* = 6, ***; *p* < 0.001 vs. control, ##; *p* < 0.01, ###; *p* < 0.001 vs. vehicle).

B16 cells treated with α-MSH showed significantly increased mRNA and protein levels of MITF compared with those of the control cells. However, upon the addition of LHMW, MITF expression was significantly decreased at both the protein and mRNA levels (Figure 5).

These results suggested that LHMW may have suppressed the transcription of the tyrosinase gene family by suppressing increased MITF expression.

## 4. Discussion

Several recent reports have demonstrated the beneficial effects of probiotics such as lactic acid bacteria and bifidobacteria [28,29,30,31,32]. In addition, many findings on the beneficial effects of whey derived from fermented milk have attracted attention [33,34,35,36]. In this study, we examined the effects of LHMW on melanogenesis with the aim of exploring a potential novel function of LHMW on the skin.

We used mouse melanoma B16 cells, which have been widely applied in studies of melanogenesis. B16 cells are known to show enhanced melanin production under stimulation with α-MSH [37,38]. In this study, we confirmed that under α-MSH stimulation, the amount of intracellular melanin increased by approximately 2-fold of that in the control condition. In contrast, cotreatment with LHMW at a dose that did not induce cytotoxicity significantly suppressed the increase in melanin production stimulated by α-MSH in a concentration-dependent manner. Specifically, treatment with 3% LHMW reduced the intracellular melanin content to almost the same level as that of the control (Figure 1). These findings suggest that LHMW could be a useful cosmetic material for the development of a skin-whitening agent.

Melanogenesis is controlled by genes in the tyrosinase family, including tyrosinase, TRP1, and DCT [9]. Among them, melanin production in melanocytes is controlled mainly by activation and expression of the rate-limiting enzyme tyrosinase [39]. Upon addition of α-MSH to B16 cells, tyrosinase activity increased, which was suppressed by cotreatment with LHMW in a concentration-dependent manner (Figure 2). In addition, LHMW decreased the protein expression level of tyrosinase while simultaneously suppressing the increased protein levels of TRP1 and DCT under α-MSH stimulation (Figure 3). Based on these findings, the suppressive effect of LHMW on melanin production could have been caused by its ability to suppress the expression and activity of tyrosinase and reduce the protein expression levels of TRP1 and DCT.

The tyrosinase gene family, including tyrosinase, TRP1, and DCT, is associated with pigmentation, proliferation, and survival, and these effects are strictly controlled by MITF [26,27]. Specifically, MITF binds to the M box in the promoter region to control the expression of these tyrosinase genes [40]. Thus, MITF is a transcription factor that plays a very important role in melanogenesis [41,42]. In this study, LHMW suppressed the increase in mRNA expression levels of the tyrosinase, Trp1, and Dct genes stimulated by α-MSH in mouse B16 melanocytes (Figure 4). Therefore, suppression of the transcription of these genes appears to be associated with a reduction in the protein expression levels of the tyrosinase gene family by LHMW. We further investigated the effect of LHMW on MITF expression in B16 cells under α-MSH stimulation, which demonstrated that LHMW significantly suppressed MITF expression at both the mRNA and protein levels (Figure 5). Given these findings, the suppression of transcription and protein expression of the tyrosinase gene family by LHMW could have been caused by LHMW’s ability to decrease MITF expression.

How can LHMW suppress the expression of MITF? It has been reported that α-MSH increases the expression level of MITF, as explained below [43,44]. First, when α-MSH binds to the melanocortin-1 receptor (Mc1R), adenylate cyclase is activated to increase the intracellular cyclic 3',5'-adenosine monophosphate (cAMP) level. The increase in cAMP content in turn activates protein kinase A (PKA) and accelerates phosphorylation of the cAMP-response element-binding protein (CREB) to ultimately induce MITF expression. That is, phosphorylated CREB upregulates the transcription of MITF to ultimately increase the MITF protein expression level. To date, a material with an anti-melanin effect that targets these processes has been identified [45]. Therefore, LHMW may reduce MITF expression by suppressing adenylate cyclase, PKA, or phosphorylated CREB. We also found that LHMW reduced the mRNA level of Mc1R compared to that in cells treated with α-MSH alone (data not shown). Similar to the tyrosinase gene family, Mc1R expression is also controlled by MITF [46]. In other words, MITF controls the expression of both melanogenesis-related enzymes and their receptors. Therefore, LHMW can reduce the responsiveness to α-MSH stimulation by suppressing MITF in addition to decreasing the expression of the tyrosinase gene family.

As described above, ultraviolet irradiation enhances melanin synthesis, which is considered to be one of the causes of oxidative stress [47]. Therefore, it has been suggested that antioxidants exhibit anti-melanogenesis effects, and, in fact, the usefulness of various antioxidants as whitening agents has been clarified [48,49]. Relatedly, whey protein has been found to exhibit an antioxidant effect [50]. Based on these facts, there is a possibility that antioxidant effects may be one of the mechanisms involved in the anti-melanogenesis action of LHMW.

Although this study clarified that LHMW has a suppressive effect on melanin production, its active ingredient remains unclear. In general, whey is rich in peptides and proteins [51]. The *L. helveticus* CM4 strain used in this study has strong proteolytic activity and decomposes milk proteins to produce many peptides during fermentation [15,52]. In this study, the components contained in LHMW that reduced the expression of MITF could not be identified. Although it is still a matter of speculation, it is thought that the peptides exerted the action. In the future, it will be possible to clarify the usefulness of LHMW by searching for active ingredients and comparing with regular milk whey and other lactic acid bacteria-fermented milk whey.

In summary, our results show that LHMW suppresses melanin production, which is suggested to involve inhibition of the expression of the tyrosinase gene family by lowering the MITF expression level. LHMW may have promise as a material for cosmetics with expected clinical application in humans.

## Figures and Tables

**Table 1:** Primer sequences used for real-time PCR.

Gene	Forward	Reverse
Tyrosinase	CAAAGGGGTGGATGACCGTG	AACTTACAGTTTCCGCAGTTGA
Trp1	ATGAAATCTTACAACGTCCTCCC	GCACACTCTCGTGGAAACTGA
Dct	TTCAACCGGACATGCAAATGC	GCTTCTTCCGATTACAGTCGGG
MITF	CAAATGGCAAATACGTTACCCG	CAATGCTCTTGCTTCAGACTCT
GAPDH	GGCAAATTCAACGGCACAGT	AGATGGTGATGGGCTTCCC

## Supplementary Materials

The following are available online at https://www.mdpi.com/2072-6643/12/7/2082/s1, Figure S1: Cell viability.
