# Supplementary material for: Lactobacillus helveticus-Fermented Milk Whey Suppresses Melanin Production by Inhibiting Tyrosinase through Decreasing MITF Expression
Source: Nutrients. 2020 Jul 14;12(7):2082. doi: 10.3390/nu12072082 (PMC7400678; doi:10.3390/nu12072082)
Supplement: Supplementary file 1 [file nutrients-12-02082-s001.pdf]

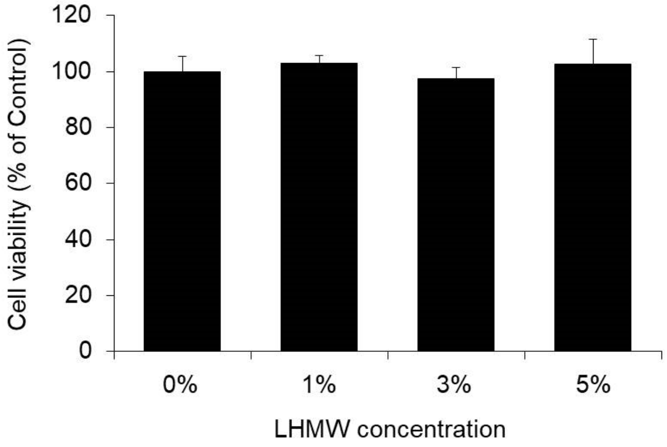

Figure 1. Cell viability. Cell viability was analyzed by the WST-1 assay, and the average value of the control was expressed as 100%.
